# Genomic characterizations of *Klebsiella variicola:* emerging pathogens identified from sepsis patients in Ethiopian referral hospitals

**DOI:** 10.1080/22221751.2024.2440494

**Published:** 2024-12-09

**Authors:** Melese Hailu Legese, Daniel Asrat, Adane Mihret, Badrul Hasan, Abraham Aseffa, Göte Swedberg

**Affiliations:** a Department of Medical Laboratory Sciences, College of Health Sciences, Addis Ababa University, Addis Ababa, Ethiopia; b Armauer Hansen Research Institute, Addis Ababa, Ethiopia; c Department of Medical Biochemistry and Microbiology, Biomedical Centre, Uppsala University, Uppsala, Sweden; d Department of Microbiology, Immunology and Parasitology, College of Health Sciences, Addis Ababa University, Addis Ababa, Ethiopia

**Keywords:** *Klebsiella variicola*, sepsis, phylogenetic relationship, antimicrobial resistance genes, plasmids, virulence genes, whole genome sequencing, Ethiopia

## Abstract

Healthcare in low- and middle-income countries is becoming problematic due to the emergence of multidrug-resistant bacteria causing serious morbidity and mortality. *Klebsiella variicola* carrying multiple antimicrobial resistance (AMR) genes were found significantly among sepsis patients in a study done between October 2019 and September 2020 at four Ethiopian hospitals located in the central (Tikur Anbessa and Yekatit 12), southern (Hawassa), and northern (Dessie) parts. Among 1416 sepsis patients, 74 *K. variicola* isolates were identified using MALDI-TOF, most of them at Dessie (*n* = 44) and Hawassa (*n* = 28) hospitals. Whole genome sequencing showed that *K. variicola* strains identified at Dessie Hospital displayed phylogenetic clonality, carried an IncM1 plasmid and the majority were ST3924. Many *K. variicola* identified at Hawassa Hospital were clonally clustered and the majority belonged to novel STs and carried IncFIB(K) and IncFII(K) plasmids concurrently. Fifty *K. variicola* carried ESBL genes while 2 isolates harboured AmpC. Other frequently found genes were *aac(3)-lla, bla*_CTX-M-15_, *bla*_TEM-1B_, *bla*_LEN2,_ *bla*_OXA-1_, *bla*_SCO-1_, *catB3*, *dfrA14*, *QnrB1*, *aac(6’)-lb-cr* and *sul2*. Virulence genes detected at both sites were *mrk operons* for biofilm formation and siderophore ABC transporter operons for iron uptake. Capsular alleles varied, with *wzi 269* at Dessie and *wzi 582* at Hawassa. The isolation of multidrug-resistant *K. variicola* as an emerging sepsis pathogen calls for strong infection prevention strategies and antimicrobial stewardship supported by advanced bacterial identification techniques.

## Background

The genus *Klebsiella* comprises various clinically relevant species such as *K. aerogenes*, *K. granulomatis*, *K. michiganensis*, *K. oxytoca*, *K. pneumoniae*, *K. quasipneumoniae*, and *K. variicola* [1,2]. *K. variicola* was first identified from plants in 2004 and separated as different from *K. pneumoniae* [3]. It is ubiquitous in the environment and common in wastewater, contaminated soil, and rivers [4]. It is widely recognized as a significant disease-causing agent in plants and less commonly in animals [5].

Recently, *K. variicola* has been considered an emerging human pathogen [2,6] and its clinical importance is growing [7] yet its virulence profile is not well described [2]. Isolation of *K. variicola* from various clinical cases was recorded in different studies [7–11]. It is described as an important cause of hospital outbreaks [12] with worsened outcomes and greater invasiveness [10]. *K. variicola* is a significant cause of sepsis which is a life-threatening disorder associated with a high rate of mortality [8,12,13]. An outbreak of neonatal sepsis due to hypervirulent multidrug-resistant *K. variicola* with a very high mortality rate was reported from Bangladesh [14].

Multidrug-resistant *K. variicola* is spreading and being identified in hospital settings [15]. *K. variicola* carrying several antibiotic resistance genes for the different classes of antibiotics including ESBL and carbapenemase genes were identified to pose a serious threat [14,16]. A case of colistin-resistant *K. variicola* was reported from a patient whose blood sample was investigated [17].

Accurate differentiation of *K. variicola* from *K. pneumoniae* using the conventional identification method [13,18] is difficult since they retain similar phenotypic characteristics [19]. The circulation of *K. variicola* in hospital settings before its first identification was documented in a study that analysed a previous collection of *K. pneumoniae* from different human samples [8]. Correct identification of *K. variicola* can be possible using advanced technologies like matrix-assisted laser desorption ionization-time of flight (MALDI-TOF) and molecular techniques [20–22].

The emergence of multidrug-resistant bacteria is complicating the provision of healthcare in low- and middle-income countries (LMIC) including Ethiopia [14]. More importantly, the identification of emerging pathogens using conventional biochemical methods is challenging in countries with resource limitations. In LMIC, data on the magnitude, antimicrobial resistance (AMR) genes, and genomic characterization of *K. variicola* that causes sepsis are scanty. Using the conventional bacterial identification method, the majority of *K. variicola* isolates identified in this study were misclassified as *K. pneumoniae* while a few of them were categorized as other *Klebsiella, Enterobacter, and Citrobacter* species. All of these isolates were reclassified correctly as *K. variicola* using MALDI-TOF. All *K. variicola* identified in the current study were isolated from patients investigated for sepsis at four teaching/referral hospitals.

## Materials and methods

A multicentre cross-sectional study was conducted between October 2019 and September 2020 among patients investigated for sepsis at four Ethiopian referral hospitals located in central, southern, and northern parts of the country. These hospitals were Tikur Anbessa Specialized Hospital (TASH) and Yekatit 12 Specialized Hospital Medical College (Y12HMC) in the central, Hawassa University Comprehensive Specialized Hospital (HUCSH) in the southern and Dessie Referral Hospital (DRH) in the northern parts of Ethiopia. The details of each hospital can be accessed from previous work [23].

All patients with suspected cases of sepsis and who sought medical service at the four hospitals were included in the study. The attending physician’s decision was applied to identify eligible patients as sepsis cases. Patients in all age groups were included however those who had been on antibiotic treatment within the preceding ten days were excluded from the study. The sociodemographic and clinical data of eligible patients were gathered using a standardized pretested questionnaire. From all hospitals, a total of 1416 clinically diagnosed cases of sepsis from different wards were enrolled in this study.

## Blood cultures, isolation, and identification of *K. variicola*

A single blood culture bottle system was processed for each patient. All blood culture bottles were incubated aerobically at 37°C for seven consecutive days and inspected daily for signs of bacterial growth. Blood samples that grew turbid before the seventh day and blood samples that were non-turbid on the seventh day were sub-cultured on blood agar (Oxoid Ltd, UK) and MacConkey agar (Oxoid Ltd, UK) at 37°C for 24 h. Bacterial identification was performed with standardized laboratory protocols and all bacteria were characterized by their colony characteristics, gram staining, and conventional biochemical tests. Triple sugar iron, indole, urea, citrate, lysine decarboxylase, motility, and malonate biochemical media were used for the identification of *Enterobacteriaceae*. All bacteria were stored at −70°C or −16°C and transported to the Armauer Hansen Research Institute and later brought to Sweden for further characterization. All bacteria were reidentified using MALDI-TOF as described in the previous publication [24].

## Antimicrobial susceptibility testing of *K. variicola*

The antimicrobial susceptibility testing (AST) of *K. variicola* was performed using disk diffusion. Each zone of inhibition was measured and interpreted as sensitive, intermediate, or resistant based on the standardized table supplied by the Clinical and Laboratory Standards Institute [25]. Using a sterile wire loop, 3–5 pure colonies were picked and emulsified in nutrient broth (Oxoid). Standard inocula were adjusted to 0.5 McFarland units and swabbed onto Muller-Hinton agar (Oxoid). Susceptibility of each isolate was tested against amikacin (30μg), ampicillin (10 µg), amoxicillin-clavulanic acid (20/10 µg), ampicillin-sulbactam (10/10 µg), aztreonam (30 µg), cefepime (30 μg), cefotaxime (30 µg), ceftriaxone (30 µg), ceftazidime (30 μg), cefuroxime (30 µg), ciprofloxacin (5μg), chloramphenicol (30 µg), doxycycline (30 µg), gentamicin (10 µg), meropenem (10 µg), piperacillin–tazobactam (100/10 µg), sulfamethoxazole-trimethoprim (1.25/23.75 µg) and tetracycline (30 µg). All antibiotics discs were OXOID products (Oxoid Ltd, UK).

## Whole genome sequencing (WGS) of *K. variicola*

All *K. variicola* (*n* = 74) were subjected to WGS. From all isolates, DNA was extracted manually using QIAamp DNA Mini Kit (QIAGEN, Germany) according to the manufacturer's instruction. After extraction, the DNA concentrations were measured with QubitTM3.0 (Thermo Scientific, MA, USA). The WGS was done at Science for Life Laboratory, Solna, Sweden. From each DNA sample (averagely 10 ng), 20μL was transferred into a 96-well WGS plate. Sequencing libraries were generated using Nextera XT (Illumina kits) and short-read sequencing was run on Illumina (HiSeq 2500) systems with a 150 bp insert size paired-end sequencing protocol at Science for Life Laboratory.

## Genome analysis

Genome assembly was done using SPAdes (version 3.9) and using the assembled genomes, Basic Local Alignment (BLAST) search for all *K. variicola* was done using BLASTN version 2.13.0 + tool at National Center for Biotechnology Information (NCBI) https://blast.ncbi.nlm.nih.gov/Blast.cgi. The acquired resistance genes and plasmid replicons carried with each *K. variicola* were identified using tools at the Center for Genomic Epidemiology (CGE) https://www.genomicepidemiology.org/. Single nucleotide polymorphism (SNP) variant calling, SNP filter, site validation, and inferring the phylogeny were done using CSI Phylogeny 1.4 https://cge.cbs.dtu.dk/services/CSIPhylogeny/ [26]. Visualization of tree and metadata was done using iTOL version 6.5.2 https://itol.embl.de/userInfo.cgi [27]. Multi-locus Sequence type (MLST), capsular antigen, and virulence genes of *K. variicola* were identified using the BIGSdb-Pasteur web platform available at https://bigsdb.pasteur.fr/klebsiella/. Categories of genes encoding virulence factors were determined according to Virulence Genes Database available at http://www.mgc.ac.cn/cgi-bin/VFs/v5/main.cgi.

## Statistical analysis

The data was prepared using Microsoft Office Excel and imported to SPSS version 28 for analysis. Descriptive statistics (count, mean, percentages or frequency and standard deviation) were calculated. The frequencies of acquired AMR, plasmid replicons, and virulence genes were calculated. Chi-square was used to determine associations of sociodemographics with *K. variicola* positivity rate. A *p*-value < 0.05 was considered statistically significant.

## Ethical approval

This study was approved by the Department of Microbiology, Immunology and Parasitology Ethical Review Committee (DEREC/18/19/01-H) and Institutional Review Board (AAUMF 01–008) of College of Health Science, Addis Ababa University. It was also approved by AHRI/ALERT Ethics Review Committee (protocol number: P050/18) of the Armauer Hansen Research Institute and National Ethical Review Committee (Ref No. MoSHE//RD/14.1/690/19).

## Results

### Sociodemographic of study participants

In this study, a total of 1416 patients investigated for sepsis at the four hospitals were included. The frequency of sepsis patients at TASH, HUCSH, DRH, and Y12HMC was 501, 316, 301, and 298, respectively. The proportion of male participants was 55.3% while females were 46.7%. Patients’ ages ranged from half a day to 90 years with a mean age of 8.85 years. Detailed patient characteristics can be accessed from previous work [24].

### *Klebsiella variicola* identification, frequencies, and whole genome analysis

Among 1416 patients investigated for sepsis, a total of 74 *K. variicola* were identified. Of these, 44 were identified at DRH from the north while 28 were isolated at HUCSH from the south (Table 1). Only two cases were identified at TASH from the central part of the country while no *K. variicola* was identified at Y12HMC (Table 1). The majority of *K. variicola* (*n* = 68) were identified at the neonatal intensive care units (NICU) from patients of age ≤29 days (Table 1). Three isolates were identified from adult patients at the intensive care unit (ICU) and emergency outpatient department (EOPD) (Table 1). Almost all patients whose blood samples had *K. variicola* were inpatients (*n* = 73) and had a 1-week hospital stay duration (*n* = 66). The majority of patients had a fever (*n* = 56) while 9 patients were hypothermic (Table 1).

**Table 1.** Frequency of *Klebsiella variicola* in relation to patient sociodemographic and clinical characteristics.

Patient sociodemographic and clinical characteristics		Frequency of *K. variicola* (*n* = 74)	
		Count	Percentage
Hospital	DRH	44	59
	HUCSH	28	38
	TASH	2	3
	Y12HMC	–	–
Gender	Male	41	55
	Female	33	45
Age category	≤29 days	68	92
	30 days – ≤5 year	2	3
	>5 to < 18 year	1	1
	≥18 years	3	4
Ward	EOPD	1	1
	ICU	2	3
	NICU	68	92
	Paediatrics	3	4
Hospital stay duration	1 week	66	89
	2 weeks	8	11
Hospitalization status	Inpatient	73	99
	Outpatient	1	1
Underlining diseases	Yes	9	12
Previous admission	Yes	5	7
Patients who referred from other health facilities	Yes	23	31
Previous antibiotic treatment history	Yes	6	8
Fever	Yes	56	76
Duration of fever category	Up to 3 days	63	85
	4–6 days	8	11
	7 days and above	3	4
Hypothermia	Yes	9	12
Abnormal Heart rate	Yes	45	61
Abnormal WBC count	Yes	50	68
Tachypnoea	Yes	57	77
Tachycardia	Yes	39	53
Apnea	Yes	9	12
Vomiting	Yes	12	17
Irritability	Yes	6	8
Hypotension	Yes	5	7
Cough	Yes	4	6
Prostration	Yes	1	1
Skin rash	Yes	1	1
Bleeding tendency	Yes	3	4
Consciousness/lethargy	Yes	8	11
Seizure/convulsion	Yes	14	19
Headache	Yes	4	6
Body weakness	Yes	1	1
Positive meningeal signs	Yes	2	3

TASH, Tikur Anbessa Specialized Hospital; Y12HMC, Yekatit 12 Specialized Hospital Medical College; DRH, Dessie Referral Hospital, HUCSH, Hawassa University Comprehensive Specialized Hospital; * Patients who were transferred from other healthcare facilities to the study sites.

At each study site, most *K. variicola* isolates were initially classified as *K. pneumoniae* while five of them were typed as other *Klebsiella, Enterobacter* and *Citrobacter* species using the conventional method. Using MALDI-TOF, all isolates were classified as *K. variicola* and all were confirmed using whole genome sequencing.

### Antimicrobial susceptibility patterns of *K. variicola*

The antibiotic susceptibility patterns of all *K. variicola* isolates were determined using disk diffusion (Table 2). Many were resistant to ampicillin, aztreonam, ceftazidime, cefotaxime, ceftriaxone, cefepime, cefuroxime, and gentamicin (Table 2). A few of the isolates were sensitive against all antibiotics other than ampicillin (Table 2 and Supplementary Table 1).

**Table 2.** Antimicrobial susceptibility patterns and frequencies and distributions of antimicrobial resistance genes carried by *K. variicola* identified from sepsis patients at Ethiopian referral hospitals.

Class of antibiotics	Antibiotics tested	AST pattern	Frequency *n* (%)	AMR genes detected		AMR genes distribution per hospitals			
						Total *n* (%)	DRH *n* (%)	HUCSH *n* (%)	TASH *n* (%)
Aminoglycosides	AMK	I	14(19)	Aminoglycoside acetyltransferases	*aac(3)-lla*	49(66.2)	20 (45)	27(96)	2(100)
		S	60(81)		*aac(3)-lle*	1(1.4)	1(2)	–	–
	GEN	I	1(1)	Aminoglycoside phosphotransferases	*aph(3”)-lb*	28(37.8)	1(2)	25(89)	2(100)
		R	52(70)		*aph(6)-ld*	28(37.8)	1(2)	25(89)	2(100)
		S	21(28)	Aminoglycoside adenyltransferase	*aadA2*	1(1.4)	1(2)	–	–
					*aadA5*	1(1.4)	–	–	1(50)
					*aadA16*	1(1.4)	1(2)	–	–
				Aminoglycoside nucleotidyltransferase	*ant(3”)–la*	2(2.7)	–	2(7)	–
b-lactams	AMP	I	1(1)	b-lactamases	*bla*_CTX–M–3_	2(2.7)	2(5)	–	–
		R	73(99)		*bla*_CTX–M–15_	48(64.9)	20 (45)	26(2)	2(100)
	AMC	I	21(28)		*bla*_TEM–1B_	48(64.9)	19(43)	27(96)	2(100)
		R	23(31)		*bla*_TEM–1C_	1(1.4)	1(2)	–	–
		S	30(41)		*bla*_SHV–187_	1(1.4)	1(2)	–	–
	SAM	I	8(11)		*bla*_LEN–2_	41(55.4)	41(93)	–	–
		R	41(55)		*bla*_LEN16_	28(37.8)	1(2)	25(89)	2(100)
		S	25(34)		*bla*_LEN18_	1(1.4)	1(2)	–	–
	ATM	I	2(3)		*bla*_LEN24_	2(2.7)	–	2(7)	–
		R	49(66)		*bla*_LEN25_	2(2.7)	–	2(7)	–
		S	23(31)		*bla*_DHA–1_	2(2.7)	–	2(7)	–
	FEP	I	3(4)		*bla*_SCO–1_	19(25.7)	19(43)	–	–
		R	48(65)		*bla*_OXA–1_	28(37.8)	1(2)	25(89)	2(100)
		S	23(31)						
	CTX	R	52(70)						
		S	22(30)						
	CRO	R	52(70)						
		S	22(30)						
	CAZ	R	52(70)						
		S	22(30)						
	CXM	R	53(72)						
		S	21(28)						
	MEM	R	1(1)						
		S	73(99)						
	TZM	I	6(8)						
		R	3(4)						
		S	65(88)						
Quinolones and fluoroquinolones	CIP	R	29(39)	Quinolones resistace genes	*aac(6’)-lb-cr*	30(40.5)	2(5)	26(2)	2(100)
		S	45(61)		*QnrB1*	26(35.1)	–	24(86)	2(100)
					*QnrB4*	2(2.7)	–	2(7)	–
					*qnrS1*	3(4.1)	1(2)	2(7)	-
					*OqxB*	1(1.4)	1(2)	-	-
Folate pathway antagonstis	SXT	R	28(38)	Sulfonamide resistance genes	*sul1*	5(6.8)	2(5)	2(7)	1(50)
		S	46(62)		*sul2*	29(39.2)	2(5)	25(89)	2(100)
				Trimethoprim resistance genes (dihydrofolate reductase (drf))	*dfrA7*	2(2.7)	–	2(7)	–
					*dfrA12*	2(2.7)	1(2)	1(4)	–
					*dfrA14*	30(40.5)	1(2)	27(96)	2(100)
					*dfrA17*	1(1.4)	–	–	1(50)
					*dfrA27*	1(1.4)	1(2)	–	–
Phenicol	C	I	1(1)	Chloramphenicol acetyltransferase	*catA1*	3(4.1)	–	2(7)	1(50)
		R	4(5)		*catB3*	28(37.8)	1(2)	25(89)	2(100)
		S	69(93)						
				Macrolides resistance genes	*mph(A)*	2(2.7)	1(2)	–	1(50)
					*mdf(A)*	1(1.4)	–	–	1(50)
				Rifampicin resistance gene	*ARR3*	1(2)	2(7)	–	
				Disinfectant resistance genes	*OqxB*	1(1.4)	1(2)	–	–
					*qacE*	5(6.8)	2(5)	2(7)	1(50)
					*sitABCD*	1(1.4)	–	–	1(50)

AST, antimicrobial susceptibility testing; S, sensitive; R, resistance; I, intermediate; AMK –amikacin; AMP, ampicillin; AMC, amoxicillin/clavulanate; SAM, ampicillin-sulbactam; ATM, aztreonam; FEP, cefepime; CTX, cefotaxime; CRO, ceftriaxone; CAZ, ceftazidime; CXM, cefuroxime; CIP, ciprofloxacin; C, chloramphenicol; DO, doxycycline; GEN, gentamicin; MEM, meropenem; TZP, piperacillin/tazobactam; SXT, trimethoprim-sulfamethoxazole; TE, tetracycline; TASH, Tikur Anbessa Specialized Hospital; DRH, Dessie Referral Hospital, HUCSH, Hawassa University Comprehensive Specialized Hospital.

Many *K. variicola* identified at HUCSH in the south showed resistance against multiple antibiotics however they were mostly sensitive to amikacin, chloramphenicol, meropenem, and piperacillin-tazobactam (TZP). Only one isolate was sensitive to all classes of antibiotics except ampicillin (Table 2). The majority of *K. variicola* identified at DRH showed resistance to most cephalosporins however they were sensitive to other classes of antibiotics (Table 2). Among the 44 *K. variicola* identified at DRH, 15 of them showed sensitivity against all antibiotics other than ampicillin (Table 2). Comparatively, *K. variicola* isolates identified at HUCSH were more resistant than those identified at DRH. The two *K. variicola* identified at TASH showed a similar pattern of resistance against multiple antibiotics and they were sensitive against meropenem and TZP (Table 2). Only one *K. variicola* isolate showed phenotypic resistance to meropenem (Table 2) however it was classified as sensitive against meropenem, imipenem, and ertapenem using E-test and micro-broth dilution.

### AMR genes carried by *K. variicola*

Among all *K. variicola* (*n* = 74), at least one resistance gene against β-lactams (*n* = 73), aminoglycosides (*n* = 49), phenicols (*n* = 30), trimethoprim (*n* = 31), macrolides (*n* = 2), quinolones (*n* = 31), tetracyclines (*n* = 29), sulphonamides (*n* = 31), disinfectants (*n* = 5) and rifampicin (*n* = 3) was detected. *aac(3)-lla* (*n* = 49)*, bla*_CTX-M-_15 (*n* = 48), *bla*TEM_-1B_ (*n* = 48) and *bla*_LEN2_ (*n* = 41) were the most commonly encoded resistance genes. *aph(3”)-lb*, *aph(6)-ld*, *bla*_LEN16_, *bla*_OXA-1_, *catB3, dfrA14*, *aac(6’)-lb-cr*, *tet(A)* and *sul2* were detected with similar proportion (Table 2). Only one isolate did not harbour any AMR genes. Of all *K. variicola*, 50 of them carried extended-spectrum-beta-lactamase (ESBL) genes while 2 had AmpC genes. All isolates were devoid of carbapenemase genes (Table 2).

At DRH, the most frequently detected resistance genes were *aac(3)-lla, bla*_CTX-M-15_, *bla*_TEM-1B_, *bla*_LEN-2_, and *bla*_SCO-1_ (Table 2). All cases of *bla*_LEN-2_ and *bla*_SCO-1_ were detected at DRH while one case of *bla*_OXA-1_ was identified. At this hospital, there was no detection of genes encoding tetracycline resistance (Table 2). At HUCSH, *K. variicola* was found carrying *aac(3)-lla*, *aph(3”)-lb*, *aph(6)-ld*, *aac(6’)-lb-cr*, *bla*_CTX-M-15_, *bla*_TEM-1B,_ *bla*_LEN16_, *bla*_OXA-_1, *catB3*, *dfrA14*, *QnrB1*, *tetA* and *sul2* as the most frequent genes. All cases of *bla*_LEN16_ were identified at HUCSH while no *bla*_SHV_ cases at all were found (Table 2). Almost all cases of *bla*_OXA-_1 were detected at HUCSH (Table 2).

### Genetic diversity and phylogenetic structure of *K. variicola* isolates

**Figure 1.**
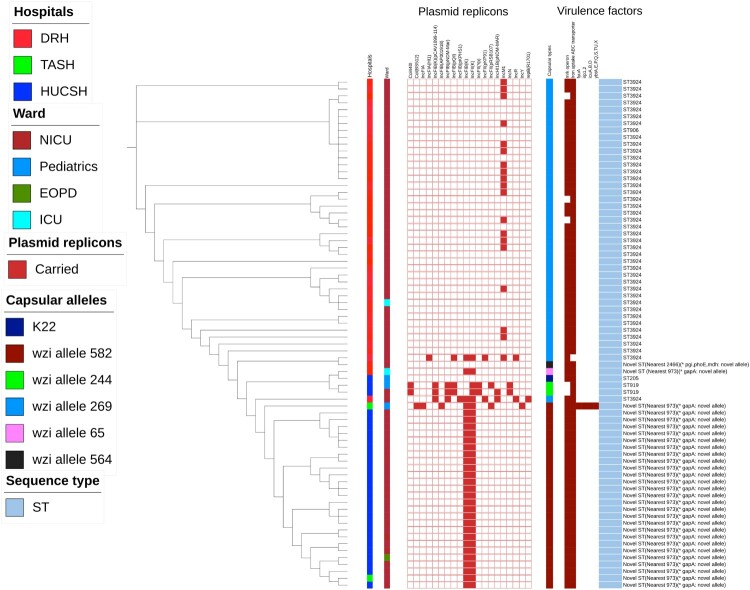
Maximum-likelihood tree generated from the core genome of 74 *K. variicola* isolates identified from sepsis patients in Ethiopian referral hospitals. TASH, Tikur Anbessa Specialized Hospital; DRH, Dessie Referral Hospital, HUCSH, Hawassa University Comprehensive Specialized Hospital: NICU, neonatal intensive care unit; EOPD, emergency outpatient department; ICU, intensive care unit; ST, sequence type; mrk operon includes mrkA, mrkB, mrkC, mrkD, mrkF, mrkH, mrkI, and mrkJ genes.

The core genome maximum likelihood tree constructed for the 74 *K. variicola* showed that distinct clones were circulating at the three hospitals (Figure 1). *K. variicola* strains identified at DRH showed clonality in the tree while a few isolates were distinct (Figure 1). The majority of *K. variicola* clones detected at DRH were ST3924 while the rest were ST906 and novel STs (Figure 1). Almost all *K. variicola* clones at DRH were identified at the NICU while only two strains were detected in its ICU department from adult patients (Figure 1).

Most of the *K. variicola* identified at HUCSH were clonally diverse, though some were identical (Figure 1). Unlike clones detected at DRH, the sequence types of many *K. variicola* identified at HUCSH were not distinctly determined and only three of them were identified as ST919 and ST205. The majority of *K. variicola* identified at HUCSH were noted as novel sequence types with a novel *gabA* allele. The majority of *K. variicola* clones at HUCSH were identified at NICU while four strains were isolated in its paediatrics and EOPD departments.

From the central part of the country, only two *K. variicola* stains were identified at TASH while there was no isolation of *K. variicola* at Y12HMC (Figure 1). The two *K. variicola* identified at TASH were isolated at its NICU and paediatrics department. They were unrelated clones and of novel sequence types (Figure 1). While one of the *K. variicola* showed similarity with clones identified at HUCSH, the other *K. variicola* identified at TASH was unrelated to all other *K. variicola* identified from all hospitals (Figure 1). The *K. variicola* strains identified at the three hospitals were collected at different time points over the collection period.

The SNP pair count matrix revealed that two major clonal groups within the sequence types circulated at study sites (Supplementary Figure 1). The first group is located in the upper-right quadrant of the matrix (white areas), with internal SNP differences ranging from 0 to 30, indicating recent clonal expansion or dissemination at DRH (Supplementary Figure 1). The second group, positioned in the lower-right quadrant (light pink area), shows a higher internal SNP variation (18,000–20,000 SNPs), suggesting some level of genetic divergence within this clonal population (Novel ST(Nearest 973)) identified at HUCSH (Supplementary Figure 1). Additionally, the novel ST (DRH_Novel ST(Nearest 2466)) appears in the matrix with a burgundy colour with a substantial SNP difference (approximately 60,000 SNPs) from other sequence types may indicate a more distantly related lineage or an evolutionary separation (Supplementary Figure 1).

### Plasmid replicons carried by *K. variicola*

*K. variicola* identified at the three hospitals had different patterns of plasmid contents although most of them carried similar types of plasmids within each hospital (Figure 1). Among the 44 *K. variicola* identified at DRH, 18 of them had at least one plasmid replicon while the majority were devoid of any plasmid contents (Figure 1). Most of the *K. variicola* identified at DRH carried IncM1 while one strain was found carrying multiple plasmid replicons together with IncM1 (Figure 1). Only one *K. variicola* identified at DRH carried several plasmids devoid of IncM1 (Figure 1). The most similar plasmid with IncM1 in the GenBank was *Klebsiella pneumoniae plasmid pMU407* (GenBank accession number U27345). Many AMR genes carried by *K. variicola* were located on the same 80.4 kb contig as the IncM1.

Among the 28 *K. variicola* isolated at HUCSH, all except one had at least one plasmid replicon. The majority harboured IncFIB(K) and IncFII(K) concurrently (Figure 1). *Klebsiella pneumoniae strain ST258 plasmid pKPN-IT* (GenBank accession number JN233704) was a similar plasmid with IncFIB(K) while IncFII(K) was matched with *Klebsiella pneumoniae subsp. pneumoniae MGH 78578 plasmid pKPN3* (GenBank accession number JN233704). Only two *K. variicola* isolated at HUCSH carried multiple plasmids: IncFIA(HI1), IncFIB(K), IncFIB(pQil), IncFII(K), IncFII(pKP91), IncM1 and IncR (Figure 1).

At TASH, one isolates carried IncFIB(K) and IncFII(K) while the other isolate harboured Col(BS512), IncFIA, IncFIB(AP001918), IncFIB(K), and IncY together with IncFIB(K) and IncFII(K) (Figure 1). Only a few isolates identified at each hospital were found to carry multiple plasmid replicons.

### Virulence factors and capsular types of *K. variicola*

All *K. variicola* were scanned for genes for virulence factors: biofilm formation, efflux pump, immune evasion, iron uptake, nutritional factor, regulation, secretion system, serum resistance, and toxin production. Almost all *K. variicola* had virulence factor genes for biofilm formation and iron uptake but no genes for other categories of virulence factors (Figure 1). The majority of *K. variicola* identified at all hospitals had the *mrk* operon (*mrkA, mrkB, mrkC, mrkD, mrkF, mrkH, mrkI,* and *mrkJ* genes) for biofilm formation. Regarding siderophore genes, all *K. variicola* identified at the three hospitals had *iron ABC transporter* operons (*kfuA*, *kfuB*, *kfuC* genes) (Figure 1). Using the BIGSdb-Pasteur web platform, *mrkC* and *kfuB* from *mrk* and *iron ABC transporter* operons were not detected. Of the two *K. variicola* identified at TASH from central Ethiopia one also carried *fyuA, irp1, irp2, icuA, icuB, icuD, ybtA, ybtE, ybtP, ybtQ, ybtS, ybtT, ybtU,* and *ybtX*. The other *K. variicola* isolated at TASH had similar virulence genes as most *K. variicola* identified at DRH and HUCSH (Figure 1).

All *K. variicola* were determined for their capsular types (*K-types*); however, it was not possible to categorize them into known K-antigens using the available databases (Figure 1). Almost all *K. variicola* identified at DRH had a similar pattern of capsular alleles (*wzi allele 269*) while three isolates had *wzi* alleles *65*, *508*, and *564*. Among 28 *K. variicola* identified at HUCSH, 24 had a similar capsular type with a *wzi* allele *582* while two had a *wzi* allele *244*. One *K. variicola* isolated at HUCSH had a similar capsular allele (*wzi* allele *269*) to the *K. variicola* isolates detected at DRH. One *K. variicola* identified at HUCSH had a known capsular type *K22*. It was not possible to determine the somatic (O) antigens of all *K. variicola* strains due to the unavailability of a database.

## Discussion

*K. variicola* is emerging as a human pathogen and its medical implication is mounting globally [14,19] since its first identification from plants in 2004 [3]. However, in most cases, human infections due to *K. variicola* are underrated and its epidemiology is not well known because of the misidentification [2,28]. This is the first report of *K. variicola* in Ethiopia among patients investigated for sepsis in the central, southern, and northern parts, though several clinical cases of *K. variicola* have been reported across the globe [1,7–11,13–15]. In the current study, *K. variicola* was identified as a leading sepsis-causing etiology at DRH in the north and HUCSH in the south, however, it was rarely detected in hospitals in the central part. Isolation of *K. variicola* among sepsis patients was evident in a study from Bangladesh [14]. Another study from Sweden showed that *K. variicola* was a leading cause of bloodstream infections in a specified geographical location; the Stockholm area [13]. The current study showed that *K. variicola* emerged as a significant sepsis pathogen that calls for implementing better infection prevention strategies. The variable magnitude and sources of *K. variicola* between hospitals in different parts demand further study.

Similar to previous studies done elsewhere [13,29,30], all *K. variicola* isolates identified in the current study were initially mistyped as *K. pneumoniae* using the conventional method. This finding suggests the need to implement advanced bacterial identification methods; however, it might be challenging for resource-limiting settings including Ethiopian hospitals’ microbiology laboratories. In such resource constraints, the introduction of adonitol (a distinctive characteristic from *K. pneumoniae*) in the routine biochemical identification method could support the clear separation of *K. variicola* (do not utilize adonitol) as an earlier study showed [3]. However, further studies will be necessary before implementation countrywide.

In this study, many *K. variicola* identified carried resistance genes against all classes of antibiotics. The emergence of multidrug-resistant *K. variicola* was reported in Bangladesh [14] and China [16,31]*.* The carriage of several β-lactamase genes enabled *K. variicola* isolates to make most cephalosporins (including the first-line regime) ineffective phenotypically. The frequent isolation of ESBL-producing *K. variicola* was concurrent with other studies done elsewhere [6,13,14,32]. The rise of multiple AMR genes, mainly ESBL, carried by *K. variicola* in clinical settings is worrisome coupled with identification difficulties, mainly an issue for LMIC. The emergence of carbapenemase genes encoding *K. variicola* was reported in Bangladesh [14], China [16], and The United Kingdom [33], however, all *K. variicola* isolates obtained in this study were devoid of any carbapenemase gene, a fact that has clinical importance for the country. *K. variicola* isolates identified at HUCSH were resistant to more of the antibiotics tested as compared with those identified at DRH. The spread of MDR strains varies geographically however further investigation is necessary to assess factors contributing to its high magnitude in some hospitals and a lower identification rate in other hospitals within a single country so that site-specific effective control mechanisms can be implemented.

The phylogeny analysis revealed that two groups of *K. variicola* isolates were circulating in Ethiopian referral hospitals: ST3924 at DRH and novel STs at HUSCH and TASH. This first knowledge of the different *K. variicola* clones circulating in Ethiopian hospitals located in different parts has public health significance that calls for site-specific interventions.

Interestingly, many *K. variicola* isolates identified at DRH carried an IncM1 plasmid similar to the one carried by *Pantoea dispersa* identified at this hospital [34]. This shows that different species of *Enterobacterales* are sharing plasmids that are circulating in the hospital. On the contrary, *K. variicola* identified at HUSCH carried IncFIB(K) and IncFII(K) plasmids concomitantly. The carriage of plasmids contributes to the evolution of bacteria by letting them adapt to environments and confer antibiotic resistance [35]. Many *K. variicola* had virulence factors for iron uptake and adhesion/biofilm formation simultaneously. These virulence factors were described in another study [14]. The capsular operon investigation showed different *wzi* alleles between *K. variicola* identified at DRH in the north and those isolated at HUSCH in the south. The findings of these unique capsular alleles coupled with the limitations of platforms to detect virulence genes that have been present in known operon groups suggested the need for further molecular characterization of *K. variicola*.

## Limitations of the study

The identification and report of MDR *K. variicola* strains using advanced bacterial characterization methods was the strength of the study. However, insufficient information on the risk factors associated with the occurrence of multidrug-resistant *K. variicola* isolates is the limitation of this study.

## Conclusion

*K. variicola* was identified as an emerging pathogen in Ethiopian referral hospitals and as a primary sepsis etiology at Dessie and Hawassa Hospitals. Two clonally different groups of *K. variicola* circulated in the north and south. These clones carried ESBLs and several other types of AMR genes. These findings call for the implementation of strong infection prevention strategies and antimicrobial stewardship in clinical settings. All *K. variicola* isolates were mischaracterized using the ordinary biochemical identification method hence advanced bacterial identification techniques are necessary for better identification.

## Supplementary Material

Supplementary Table 1.docx

Supplementary Figure 1.jpg

## Data Availability

The genomic sequence data were submitted to the National Center for Biotechnology Information (BioProject ID: PRJNA787062).
