# Supplementary material for: Genomic characterizations of Klebsiella variicola: emerging pathogens identified from sepsis patients in Ethiopian referral hospitals
Source: Emerg Microbes Infect. 2024 Dec 9;14(1):2440494. doi: 10.1080/22221751.2024.2440494 (PMC11656759; doi:10.1080/22221751.2024.2440494)
Supplement: Supplementary Table 1.docx [file TEMI_A_2440494_SM1975.docx]

Supplementary Table 1. Antimicrobial susceptibility patterns of *K. variicola* identified from sepsis patients at Ethiopian referral hospitals*.*

| **Hospital** |  | **Bacteria** | **AMK** | **AMP** | **AMC** | **SAM** | **ATM** | **FEP** | **CTX** | **CRO** | **CAZ** | **CXM** | **CIP** | **C** | **DO** | **GEN** | **MEM** | **TZP** | **SXT** | **TE** |
| --- | --- | --- | --- | --- | --- | --- | --- | --- | --- | --- | --- | --- | --- | --- | --- | --- | --- | --- | --- | --- |
|  | HUCSH | *K.variicola* | S | R | R | R | R | R | R | R | R | R | R | S | R | R | S | S | R | R |
|  | HUCSH | *K.variicola* | I | R | R | R | R | R | R | R | R | R | R | S | R | R | S | S | R | R |
|  | HUCSH | *K.variicola* | I | R | R | R | R | R | R | R | R | R | R | S | R | R | S | S | R | R |
|  | HUCSH | *K.variicola* | I | R | R | R | R | R | R | R | R | R | R | S | R | R | S | S | R | R |
|  | HUCSH | *K.variicola* | I | R | I | R | R | R | R | R | R | R | R | S | I | R | S | S | S | S |
|  | HUCSH | *K.variicola* | I | R | R | R | R | R | R | R | R | R | R | S | R | R | S | I | R | R |
|  | HUCSH | *K.variicola* | S | R | R | R | R | R | R | R | R | R | R | R | R | R | S | S | R | R |
|  | HUCSH | *K.variicola* | S | R | R | R | S | I | R | R | R | R | R | I | R | R | S | S | R | R |
|  | HUCSH | *K.variicola* | S | R | I | R | R | R | R | R | R | R | R | S | I | R | S | S | R | R |
|  | HUCSH | *K.variicola* | S | R | I | R | R | R | R | R | R | R | R | S | R | R | S | S | R | R |
|  | HUCSH | *K.variicola* | S | R | R | R | R | R | R | R | R | R | R | R | I | R | S | S | R | R |
|  | HUCSH | *K.variicola* | S | R | I | R | R | R | R | R | R | R | R | S | R | R | S | S | R | R |
|  | HUCSH | *K.variicola* | I | R | R | R | R | R | R | R | R | R | R | S | R | R | S | I | S | R |
|  | HUCSH | *K.variicola* | S | R | R | I | R | R | R | R | R | R | R | S | R | R | S | I | S | R |
|  | HUCSH | *K.variicola* | S | R | I | R | R | R | R | R | R | R | R | S | I | R | S | S | R | R |
|  | HUCSH | *K.variicola* | S | R | R | R | R | R | R | R | R | R | R | S | R | R | S | S | R | R |
|  | HUCSH | *K.variicola* | S | R | R | S | R | R | R | R | R | R | R | S | R | R | S | I | R | R |
|  | HUCSH | *K.variicola* | S | R | R | R | R | R | R | R | R | R | R | S | R | R | S | S | R | R |
|  | HUCSH | *K.variicola* | I | R | I | R | R | I | R | R | R | R | R | S | R | R | S | S | R | R |
|  | HUCSH | *K.variicola* | S | R | R | R | R | R | R | R | R | R | R | S | R | R | S | S | S | R |
|  | HUCSH | *K.variicola* | I | R | R | R | R | R | R | R | R | R | R | S | R | R | S | S | R | R |
|  | HUCSH | *K.variicola* | S | R | R | R | R | R | R | R | R | R | R | S | R | R | S | S | R | R |
|  | HUCSH | *K.variicola* | S | R | R | R | R | R | R | R | R | R | R | S | R | R | S | S | R | R |
|  | HUCSH | *K.variicola* | S | R | R | R | R | R | R | R | R | R | R | S | R | R | S | I | R | R |
|  | HUCSH | *K.variicola* | I | R | R | R | R | R | R | R | R | R | R | S | R | R | S | I | R | R |
|  | HUCSH | *K.variicola* | I | R | I | R | R | R | R | R | R | R | R | S | R | R | S | S | R | R |
|  | HUCSH | *K.variicola* | I | R | R | R | R | R | R | R | R | R | R | S | R | R | S | S | R | R |
|  | HUCSH | *K.variicola* | I | R | S | S | S | S | S | S | S | S | S | S | S | S | S | S | S | S |
|  | TASH | *K.variicola* | I | R | R | R | R | R | R | R | R | R | R | R | R | R | S | S | R | R |
|  | TASH | *K.variicola* | I | R | R | R | R | R | R | R | R | R | R | R | R | R | S | S | R | R |
|  | DRH | *K.variicola* | S | R | S | S | S | S | S | S | S | S | S | S | S | S | S | S | S | S |
|  | DRH | *K.variicola* | S | R | S | R | R | R | R | R | R | R | S | S | S | R | S | S | S | S |
|  | DRH | *K.variicola* | S | R | S | S | S | S | S | S | S | S | S | S | S | S | S | S | S | S |
|  | DRH | *K.variicola* | S | R | S | S | S | S | S | S | S | S | S | S | S | R | S | S | S | R |
|  | DRH | *K.variicola* | S | R | S | S | R | S | S | S | S | S | S | S | S | S | S | S | S | S |
|  | DRH | *K.variicola* | S | R | I | R | R | R | R | R | R | R | S | S | S | R | S | S | S | S |
|  | DRH | *K.variicola* | S | R | I | S | I | S | S | S | S | S | S | S | S | S | S | S | S | S |
|  | DRH | *K.variicola* | S | R | S | S | S | S | S | S | S | S | S | S | S | S | S | S | S | S |
|  | DRH | *K.variicola* | S | R | I | R | R | R | R | R | R | R | S | S | S | R | S | S | S | S |
|  | DRH | *K.variicola* | S | R | S | S | S | S | S | S | S | S | S | S | S | S | S | S | S | S |
|  | DRH | *K.variicola* | S | R | S | S | S | S | S | S | S | S | S | S | S | S | S | S | S | S |
|  | DRH | *K.variicola* | S | R | S | S | S | R | R | R | R | R | S | S | S | R | S | R | R | R |
|  | DRH | *K.variicola* | S | R | S | S | S | S | S | S | S | S | S | S | S | S | S | S | R | R |
|  | DRH | *K.variicola* | S | R | S | S | S | S | S | S | S | S | S | S | S | S | S | S | S | S |
|  | DRH | *K.variicola* | S | R | S | S | S | S | S | S | S | S | S | S | S | S | S | S | S | S |
|  | DRH | *K.variicola* | S | R | S | S | S | S | S | S | S | S | S | S | S | S | S | S | S | S |
|  | DRH | *K.variicola* | S | R | S | S | S | S | S | S | S | S | S | S | S | I | S | S | S | S |
|  | DRH | *K.variicola* | S | R | S | S | S | S | S | S | S | S | S | S | S | S | S | S | S | R |
|  | DRH | *K.variicola* | S | R | S | I | R | R | R | R | R | R | S | S | S | R | S | S | S | S |
|  | DRH | *K.variicola* | S | R | I | I | R | R | R | R | R | R | S | S | S | R | S | S | S | S |
|  | DRH | *K.variicola* | S | R | S | R | R | R | R | R | R | R | S | S | S | R | R | R | R | S |
|  | DRH | *K.variicola* | S | R | S | S | I | S | S | S | S | S | S | S | S | S | S | S | S | S |
|  | DRH | *K.variicola* | S | R | S | S | S | S | S | S | S | S | S | S | S | S | S | S | S | S |
|  | DRH | *K.variicola* | S | R | I | R | R | R | R | R | R | R | S | S | S | R | S | S | S | S |
|  | DRH | *K.variicola* | S | R | S | S | S | S | S | S | S | S | S | S | S | S | S | S | S | S |
|  | DRH | *K.variicola* | S | R | R | I | S | I | S | R | R | R | S | S | S | S | S | S | S | S |
|  | DRH | *K.variicola* | S | R | I | R | R | S | R | R | R | R | S | S | S | R | S | S | S | S |
|  | DRH | *K.variicola* | S | R | I | R | R | R | R | R | R | R | S | S | S | R | S | S | S | S |
|  | DRH | *K.variicola* | S | R | I | S | S | S | R | S | S | R | S | S | S | R | S | S | S | S |
|  | DRH | *K.variicola* | S | R | S | S | S | S | S | S | S | S | S | S | S | S | S | S | S | S |
|  | DRH | *K.variicola* | S | R | I | R | R | R | R | R | R | R | S | S | S | R | S | S | S | S |
|  | DRH | *K.variicola* | S | R | I | R | R | R | R | R | R | R | S | S | S | R | S | S | S | S |
|  | DRH | *K.variicola* | S | R | S | I | R | R | R | R | R | R | S | S | S | R | S | S | S | S |
|  | DRH | *K.variicola* | S | R | I | R | R | R | R | R | R | R | S | S | S | R | S | S | S | S |
|  | DRH | *K.variicola* | S | R | S | R | S | R | R | R | R | R | S | S | S | S | S | S | S | S |
|  | DRH | *K.variicola* | S | R | S | R | R | R | R | R | R | R | S | S | S | R | S | S | S | S |
|  | DRH | *K.variicola* | S | R | I | R | R | R | R | R | R | R | S | S | S | R | S | S | S | S |
|  | DRH | *K.variicola* | S | R | S | S | S | S | S | S | S | S | S | S | S | S | S | S | S | S |
|  | DRH | *K.variicola* | S | R | S | R | R | R | R | R | R | R | S | S | S | R | S | S | S | S |
|  | DRH | *K.variicola* | S | R | S | S | S | S | S | S | S | S | S | S | S | S | S | S | S | S |
|  | DRH | *K.variicola* | S | R | I | I | R | R | R | R | R | R | S | S | S | R | S | S | S | S |
|  | DRH | *K.variicola* | S | R | S | I | R | R | R | R | R | R | S | S | S | R | S | S | S | S |
|  | DRH | *K.variicola* | S | R | S | I | R | R | R | R | R | R | S | S | S | R | S | S | S | S |
|  | DRH | *K.variicola* | S | R | I | S | R | R | R | R | R | R | S | S | S | R | S | R | S | S |

*S-sensitive; R – resistance; I – intermediate; AMK –amikacin; AMP – ampicillin; AMC – amoxicillin/clavulanate; SAM – ampicillin-sulbactam; ATM – aztreonam; FEP – cefepime; CTX – cefotaxime; CRO – ceftriaxone; CAZ – ceftazidime; CXM – cefuroxime; CIP – ciprofloxacin; C – chloramphenicol; DO – doxycycline; GEN – gentamicin; MEM – meropenem; TZP – piperacillin/tazobactam; SXT – trimethoprim-sulfamethoxazole; TE – tetracycline*
