# Supplementary figures and images for: Genomic characterizations of Klebsiella variicola: emerging pathogens identified from sepsis patients in Ethiopian referral hospitals
Source: Emerg Microbes Infect. 2024 Dec 9;14(1):2440494. doi: 10.1080/22221751.2024.2440494 (PMC11656759; doi:10.1080/22221751.2024.2440494)

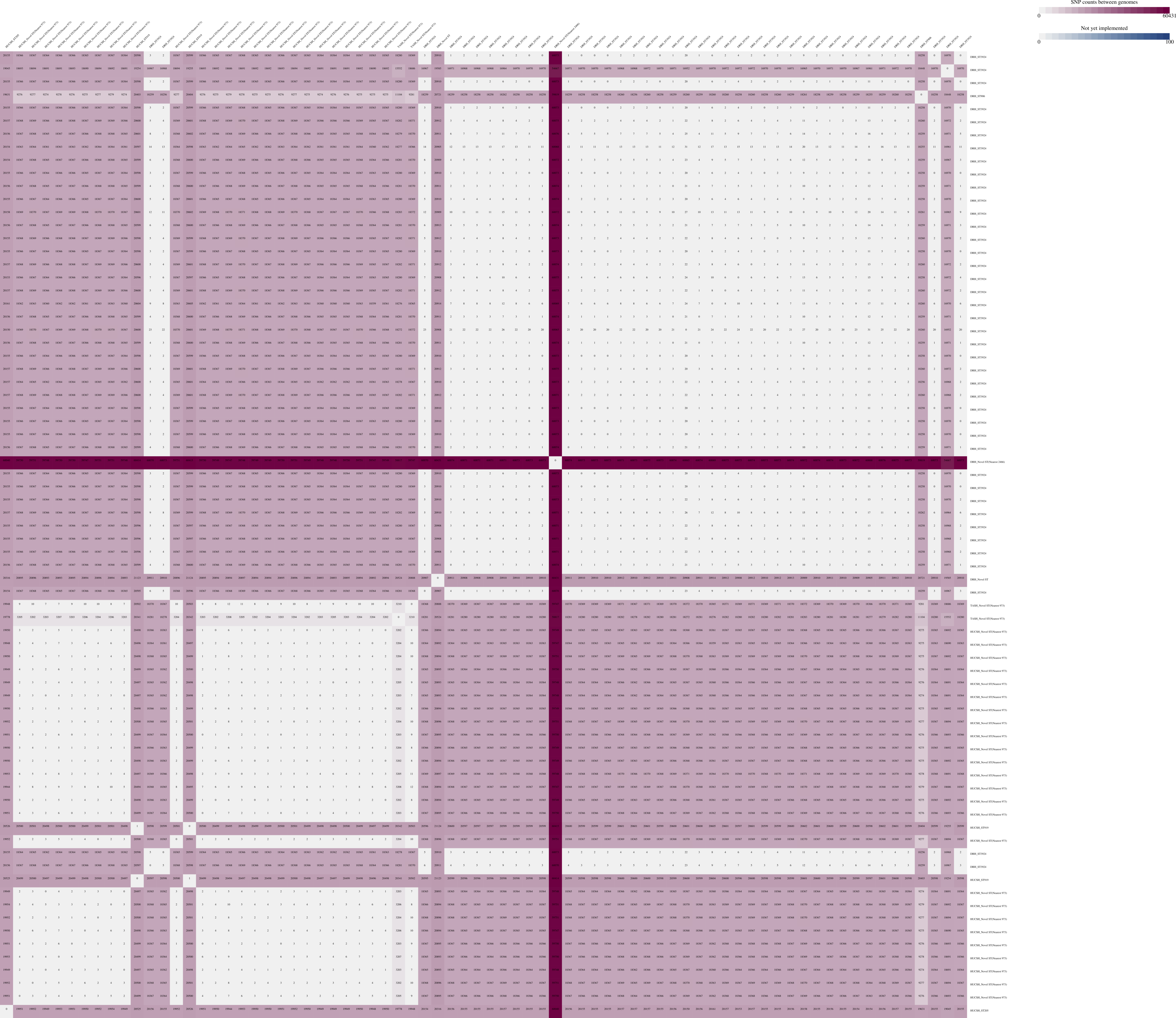

Supplement: Supplementary Figure 1.jpg [file TEMI_A_2440494_SM1974.jpg]
